# Concerns Regarding the Design, Methodology, and Potential Biases in the Türkiye Heart Failure (TURK‐HF) Registry

**DOI:** 10.1002/clc.70360

**Published:** 2026-05-28

**Authors:** Çağrı Zorlu

**Affiliations:** ^1^ Department of Cardiology Tokat Gaziosmanpasa University Hospital Tokat Turkey

Dear Editor,

I read with great interest the rationale and design paper of the Türkiye Heart Failure (TURK‐HF) Registry by Kocabas et al., published in Clinical Cardiology [1]. This national, multicenter, prospective observational study aims to provide valuable real‐world insights into the sociodemographic and clinical characteristics, management strategies, and outcomes of patients with heart failure (HF) across ejection fraction categories in Türkiye. While we commend the authors' efforts to address important knowledge gaps in a middle‐income country, we have several methodological, conceptual, and interpretive concerns.

The manuscript describes the enrollment of unselected patients with HF, regardless of ejection fraction, and emphasizes representativeness. However, participation was voluntary among 46 investigators from 38 centers, predominantly university and training hospitals, with no financial compensation provided. Voluntary participation in registries frequently introduces selection bias, as motivated centers and physicians are more likely to enroll, potentially over‐representing academic settings and under‐representing community or rural practices [2]. Although study sites were selected according to NUTS classification and Health Statistics Yearbook data, the heavy concentration in major cities such as Izmir and Istanbul may limit the reflection of Türkiye's diverse population and healthcare access disparities.

The frailty assessment relies on the Canadian Study of Health and Aging Clinical Frailty Scale based on participating physicians' clinical judgment. Without mention of standardized training or inter‐rater reliability assessment across multiple centers, this approach carries risks of subjectivity. Similarly, the detailed treatment assessment algorithm for guideline‐directed medical therapy (GDMT) is innovative but may be prone to social desirability bias, as physicians and patients provide rationales for non‐use. Notably, the paper states that initiation, up‐titration, and maintenance of therapies remain beyond the registry's scope and are the physicians' responsibility, which appears somewhat inconsistent with the ambitious goals of identifying implementation barriers.

The adoption of Dr. Korkut Boratav's class‐based socioeconomic status (SES) framework is presented as a distinctive feature. While sociologically valuable, its application in a clinical HF registry lacks prior validation in this context and may complicate international comparisons, which typically rely on standardized metrics such as income, education, and insurance status [3].

Regarding HF with preserved ejection fraction (HFpEF), the integration of the H2FPEF score as a decision support tool is promising. However, previous studies in Turkish populations have shown conflicting results concerning its diagnostic performance, raising concerns about potential misclassification in the presence of high comorbidity burden [4].

Finally, while the planned use of the win ratio for composite endpoints and 6‐month follow‐up intervals is a positive aspect, the absence of independent endpoint adjudication in an observational study represents a notable limitation.

I respectfully ask the authors the following questions:

1. How do they plan to mitigate selection bias arising from voluntary participation and the predominance of tertiary centers?
2. What specific measures will ensure data quality, completeness, and consistency for echocardiographic parameters and frailty scoring across heterogeneous sites?
3. How will temporal changes in GDMT uptake be analyzed, given evolving evidence on SGLT2 inhibitors and finerenone?
4. Will subgroup analyses by region or hospital type be adequately powered?

I believe addressing these points will strengthen the registry's credibility and impact. I look forward to the publication of the baseline results.

## Funding

The author has nothing to report.

## Conflicts of Interest

The author declares no conflicts of interest.

## Data Availability

Data sharing is not applicable to this article as no data sets were generated or analyzed during the current study.
